# Modulation of Inter-kingdom Communication by PhcBSR Quorum Sensing System in *Ralstonia solanacearum* Phylotype I Strain GMI1000

**DOI:** 10.3389/fmicb.2017.01172

**Published:** 2017-06-23

**Authors:** Peng Li, Wenfang Yin, Jinli Yan, Yufan Chen, Shuna Fu, Shihao Song, Jianuan Zhou, Mingfa Lyu, Yinyue Deng, Lian-Hui Zhang

**Affiliations:** ^1^Integrative Microbiology Research Centre, Guangdong Province Key Laboratory of Microbial Signals and Disease Control, State Key Laboratory for Conservation and Utilization of Subtropical Agro-Bioresources, South China Agricultural UniversityGuangzhou, China; ^2^School of Biological and Science Technology, University of JinanJinan, China; ^3^Guangdong Innovative and Entrepreneurial Research Team of Sociomicrobiology Basic Science and Frontier Technology, College of Agriculture, South China Agricultural UniversityGuangzhou, China; ^4^Institute of Molecular and Cell BiologySingapore, Singapore

**Keywords:** bacterial wilt, non-ribosomal peptide, interaction, soil microbes, regulatory mechanism

## Abstract

*Ralstonia solanacearum* is a ubiquitous soil-borne plant pathogenic bacterium, which frequently encounters and interacts with other soil cohabitants in competition for environmental niches. Ralsolamycin, which is encoded by the *rmy* genes, has been characterized as a novel inter-kingdom interaction signal that induces chlamydospore development in fungi. In this study, we provide the first genetic evidence that the *rmy* gene expression is controlled by the PhcBSR quorum sensing (QS) system in strain GMI1000. Mutation of *phcB* could lead to significant reduction of the expression levels of the genes involved in ralsolamycin biosynthesis. In addition, both the *phcB* and *rmy* mutants were attenuated in induction of chlamydospore formation in *Fusarium oxysporum* f. *cubense* and diminished in the ability to compete with the sugarcane pathogen *Sporisorium scitamineum*. Agreeable with the pattern of QS regulation, transcriptional expression analysis showed that the transcripts of the *rmy* genes were increased along with the increment of the bacterial population density. Taken together, the above findings provide new insights into the regulatory mechanisms that the QS system involves in governing the ralsolamycin inter-kingdom signaling system.

## Introduction

*Ralstonia solanacearum* is a notorious soil-borne pathogen that causes lethal bacterial wilt of many plants around the world (Hayward, 1991). The pathogen encodes numerous virulence determinants, including extracellular polysaccharide (EPS), cell wall-degrading enzymes (CWDE), chemotaxis system, and secretion systems, which are collectively contribute to its virulence (Denny and Ryel, 1991; Arlat et al., 1992; Yao and Allen, 2006; Genin and Denny, 2012). Previous studies have outlined the sophisticated regulatory mechanisms that control the production of virulence factors in *R. solanacearum.* Among them, PhcA is a LysR family transcriptional regulator (Brumbley et al., 1993), which is located at the center of the complex regulatory network, and can directly or indirectly regulate the genes involved in production of EPS and other virulence factors (Huang et al., 1995). Along with bacterial proliferation, PhcA activity is regulated by accumulated quorum sensing (QS) signal 3-hydroxypalmitic acid methyl ester (3-OH-PAME) or (R)-methyl 3-hydroxymyristate (3-OH-MAME), which is encoded by *phcB* (Flavier et al., 1997; Kai et al., 2015). Consequently, PhcA directs the production of EPS, CWDE, and other virulence factors in a population density dependent manner. Evidence indicates that a two component system, encoded by *phcS* and *phcR* in the same operon as *phcB*, is involved in detection and response to the QS signal 3-OH-PAME (Clough et al., 1997).

*Ralstonia solanacearum* species complex is well known not only for their ability to infect a unusually broad range of host plants, but also for their wide geographic distributions and capability to live and compete for versatile and diverse habitats (Salanoubat et al., 2002; Alvarez et al., 2010). Involvement of secondary metabolites in interspecies and inter-kingdom signaling and interference between *R. solanacearum* and the other organisms in competition for environmental niches has recently attracted much attention. Genome sequencing analysis and genetic studies show that *R. solanacearum* complex has the potential to produce an array of secondary metabolites. For example, ralfuranone is known to contribute to the virulence of *R. solanacearum* strain OE1-1 (Kai et al., 2014); staphyloferrin B is a siderophore associated with iron scavenge in strain AW1 (Bhatt and Denny, 2004); the yersiniabactin-like siderophore micacocidin was identified as an anti-mycoplasma agent (Kreutzer et al., 2011). More recently, *R. solanacearum* was reported to produce ralsolamycin as an inter-kingdom signal to communicate with fungal organisms and consequently induce conserved morphological differentiation, i.e., formation of chlamydospores that are survival structures with thickened cell walls, in 34 species of fungi belonging to three taxa (Spraker et al., 2016). Ralsolamycin is produced by the non-ribosomal peptide synthetase-polyketide synthase hybrid RmyA and RmyB, which also facilitates the bacterial pathogen entry into fungal tissues (Spraker et al., 2016). It is not yet clear how the production of ralsolamycin is regulated in *R. solanacearum*.

RmyA and RmyB are the homologs of AmbB and AmbE, respectively, which we identified previously are responsible for production of IQS, an integrative QS signal associated with virulence regulation in *Pseudomonas aeruginosa* (Lee et al., 2013). Production of IQS is controlled not only by the central *las* QS system, but also influenced by phosphate depletion, which is a host stress signal commonly encountered by invading pathogens (Lee et al., 2013; Lee and Zhang, 2015). In this study, initiated by investigating the role of *rmyAB* genes in *R. solanacearum*, we found that the expression of *rmyAB* was controlled by the PhcBSR QS system. Deletion of *phcB* resulted in dramatic decreases in transcriptional expression of *rmy* genes and ralsolamycin transportation related genes, and weakened the bacterial ability to induce formation of chlamydospores in soil-borne phytopathogens *Fusarium oxysporum* f. *cubense* (FOC) strain XJZ2 (Li et al., 2014), and lost the antimicrobial activity to inhibit the growth of *Sporisorium scitamineum*.

## Materials and Methods

### Bacterial and Fungal Strains, Plasmids, and Media

The plasmids and *R. solanacearum* strains used in this study are listed in **Table 1**. *Escherichia coli* strain DH5α (Invitrogen, Carlsbad, CA, United States) was used as a host in gene cloning and vector construction. *R. solanacearum* strains were maintained at 30°C in casamino acid-peptone-glucose (CPG) plates (Hendrick and Sequeira, 1984), and cultured in CPG broth for testing CWDE activities (Yap et al., 2005), or on minimal medium (MM) agar plate for screening transformants after tri-parental mating (Hussain et al., 2008). *E. coli* was grown at 37°C in LB medium. Antibiotics were added at the following final concentrations (μg/L): kanamycin, Km (50), gentamicin, Gm (50), and rifampicin, Rif (50). The fungal strains used in this study were maintained in PDA medium unless otherwise stated.

**Table 1:** List of the bacterial and fungal strains and plasmids used in this study.

Strain/Plasmid	Relevant characteristics	Source
***R. solanacearum***		
GMI1000	Phylotype I, wild-type, Rif^r^	Salanoubat et al., 2002; ATCC^®^ BAA1114^TM^
Δ*phcB*	*phcB* deletion mutant (Gm^r^, Rif^r^)	This study
Δ*phcB*(*phcB*)	Δ*phcB* complement (Gm^r^, Km^r^, Rif^r^)	This study
Δ*rmyA*	*rmyA* deletion mutant (Gm^r^, Rif^r^)	This study
Δ*rmyB*	*rmyB* (Rif^r^y) mutant	This study
Δ*rmyAB*	*rmyAB* double mutant (Gm^r^, Rif^r^)	This study
***E. coli***		
DH5α	λ-ϕ80d*lacZ*ΔM15Δ(*lacZYA-argF*)*U169 recA1 endA1 hsdR17 supE44 thi-1 gyrA relA1*	Invitrogen
**Fungi**		
XJZ2	*F. oxysporum* f. *cubense*	Li et al., 2014
Ss17 (*MAT-1*)	Pair of mating strains of *S. scitamineum*	Yan et al., 2016
Ss18(*MAT-2*)		
**Plasmids**		
pK18mobsacB	Km^r^, suicide and narrow-broad-host vector	Schäfer et al., 1994
pBBR1MCS2	Km^r^, broad-host-range cloning vector	Kovach et al., 1995
pRK2013	Km^r^	Ditta et al., 1980
pMD18T	T vector; Amp^r^	TAKARA


### In-Frame Deletion and Complementation

The *phcB*, *rmyA*, *rmyB*, and *rmyAB* deletion mutants were generated by amplifying two DNA fragments flanking their coding sequences (using primers as described in **Table 2**), the 859 bp gentamicin resistance gene sequence (*Gen*) was added between the left and right fragments of *phcB* or *rmyA* by using the overlap primers, the left and right DNA fragments of *rmyB* were ligated without adding the *Gen* sequence. The two/three fragments were fused using the primer pair L1/R2. All the PCR procedure of this study were amplification using the high fidelity Taq polymerase (PrimeSTAR^®^ HS DNA Polymerase, Takara Bio Inc., Dalian, China), cycling conditions were set as follows: initial denaturation at 98°C for 1 min, followed by 34 cycles of denaturation at 98°C for 15 s, annealing at 60°C for 20 s, extension at 72°C for 30 s, and a final extension at 72°C for 5 min. The fusion fragments and the suicide plasmid pK18mobsacB (ATCC^®^87097^TM^) (Schäfer et al., 1994) were digested, respectively, with corresponding restriction enzymes as indicated, followed by purification by using Sangon purification kit (Sangon, Shanghai, China), and then ligated together by using T4 ligase [NEW ENGLAND BioLabs (Beijing) Ltd., China]. The ligation products were transformed into *E. coli* DH5α competent cells (Life Technologies Corporation, Beijing, China), and the bacterial cells were cultured at 37°C with shaking for 1 h. The transformants were selected in LB medium supplemented with Km and verified by PCR analysis. The plasmid constructs were introduced into *R. solanacearum* by using tri-parental mating as described previously to generate corresponding in-frame deletion mutants (Ditta et al., 1980). Mutants were selected on CPG plate containing 10% sucrose, antibiotics Gm and Rif, and then confirmed by PCR and DNA sequencing. To construct complementary strains, the DNA fragment containing 235 bp promoter sequence and ORF of *phcB* was amplified using primers *phcB*-CF and *phcB*-CR (**Table 2**). The purified PCR products were digested by required restriction enzymes, and then purified again prior to ligation with the same enzymes digested expression vector pBBR1MCS2 (Kovach et al., 1995). The ligation products were transformed into *E. coli* DH5α competent cells, and the transformants were selected on LB plate containing Km. Tri-parental mating was performed as above description, and the complemented strains were selected on MM plates supplemented with Km and Rif, and confirmed by PCR product analysis. The gel electrophoresis results of the mutants and control are provided in Supplementary Information File 1 (SI 1).

**Table 2:** Primers used in this study.

Primer name	Prime sequence	Note
*phcB*-L1	CGGGATCCCTGTTCGGCAAGTACAATCG	*Bam*HI
*phcB*-L2	TTTCCACGGTGTGCGTCCGCTGCAGCGTGATGATGGTG	
*Gen*-M1	CACCATCATCACGCTGCAGCGGACGCACACCGTGGAAA	
*Gen*-M2	GAACACGTTGACACCGGTATGGCGGCGTTGTGACAATTT	
*phcB*-R1	AAATTGTCACAACGCCGCCATACCGGTGTCAACGTGTTC	
*phcB*-R2	GCGTCGACCTCGGTGAGGCTGTGGTTGAT	*Sal*I
*phcB*-CF	CCAAGCTTGGCCTCTCCTCCAATCATCTCG	*Hin*dIII
*phcB*-CR	CGGGATCCCGACGCCGAACAGCACCTG	*Bam*HI
*rmyA*-L1	CGGGATCCTGCTGGGAAACGGGTGC	*Bam*HI
*rmyA*-L2	TTTCCACGGTGTGCGTCCGATAGTCGGTGATCGCCTTGA	
*Gen*-M1	TCAAGGCGATCACCGACTATCGGACGCACACCGTGGAAA	
*Gen*-M2	TCGATCAAGGCATGGAAGGGGCGGCGTTGTGACAATTT	
*rmyA*-R1	AAATTGTCACAACGCCGCCCCTTCCATGCCTTGATCGA	
*rmyA*-R2	GCGTCGACGCTTCACTTGGTCGTCGCTG	*Sal*I
*rmyB*-L1	CGGAATTCTGTGCAGTTCTCGGTCAAGG	*Eco*RI
*rmyB*-L2	CACCATGTATTCCGGCAAGCCCTGCATCGGCTCGTCCAA	
*rmyB*-R1	TTGGACGAGCCGATGCAGGGCTTGCCGGAATACATGGTG	
*rmyB*-R2	CCAAGCTTGTGTGCAGCCATTTCATCGA	*Hin*dIII
16S-F	CTGGAATCGCTAGTAATCG	qRT-PCR
16S-R	AGGCTAACTACTTCTGGTAA	qRT-PCR
*RSp0638*-F	TGTTCCGTTCCTTCTTC	qRT-PCR
*RSp0638*-R	GATCTTCGTAGGTGTAGC	qRT-PCR
*RSp0639*-F	CCTATCTGGCGTATCTG	qRT-PCR
*RSp0639*-R	GCTTGAGTTCCTTGAAG	qRT-PCR
*rmyA*-F	TCAAGGCAATACGACAAG	qRT-PCR
*rmyA*-R	CGTCTTCATCGGTATCTC	qRT-PCR
*rmyB*-F	GTTCTCGACTTCGTTGA	qRT-PCR
*rmyB*-R	GAAGGCACCGTATTGAT	qRT-PCR


### Co-culture Experiments and Microscopy Examination of Chlamydospore Formation

Preparation of conidial suspensions of the FOC strain XJZ2 was performed as described (Spraker et al., 2016). For co-culture experiments, *R. solanacearum* strain GMI1000 and the fungal pathogen were plated as previously described and incubated for 8 days at 28°C. When the colonies met each other, the fungi mycelia of the interaction zone were harvested and examined for FOC chlamydospore formation by using ZEISS Observer Z1 microscopy. For testing bacterial interaction with *S. scitamineum*, the *MAT-1* and *MAT-2* (Yan et al., 2016) mating mixture of *S. scitamineum* were grown on PDA plates for observation of hypha growth. To observe the dose effect of strain GMI1000 on inhibition of *S. scitamineum*, the GMI1000 (A) was cultured 24 h on the CPG plate before adding GMI1000 (B), other GMI1000 derivatives, and the *MAT-1*/*MAT-2* mating mixture of *S. scitamineum* on the CPG plate.

### RNA Preparation and Quantitative Real-Time PCR (qRT-PCR) Analysis

Cultures of *R. solanacearum* strains in CPG broth (OD_600_ = 1.5) were centrifuged and total RNA were isolated by using RNeasy Mini Kit (QIAGEN, Hilden, Germany). The contaminated genomic DNA were removed by treating with DNaseI (Takara, Dalian, China) at 37°C for 1 h, and was confirmed by PCR using the 16S primer pair and visualized on an agarose gel. The qRT-PCR experiments were performed with SuperReal PreMix Color SYBR Green, 2X, (TIANGEN BIOTECH CO. LTD, Beijing, China) on QuantStudio 6 Flex (applied biosystems by life technologies, Carlsbad, CA, United States) following the user’s guide from the manufacturer. The PCR conditions were as follows: 95°C for 15 min, followed by 40 cycles of 95°C for 15 s and 55°C for 31 s, and then the melting curve analysis was carried out to determine the specificity of PCR products. The cDNA samples of each treatment were replicated three times, and the absolute value of –ΔΔCt = –(ΔCt_1_ – ΔCt_2_) were calculated as described in the 2^-ΔΔCt^ method (Livak and Schmittgen, 2001).

### LC/MS Analysis of Ralsolamycin

To prepare ralsolamycin extracts, bacteria were inoculated in 1 litre CPG broth and cultured at 28°C for 24 h with shaking at 150 rpm (OD_600_ = 1.5). Bacterial supernatants were collected by centrifugation at 10,000 rpm for 10 min, mixed with an equal volume of ethyl acetate with shaking. The upper organic phase was collected and air dried, and the residues were dissolved in methanol for further analysis. LC/MS analysis was conducted using a Waters LC-MS system (Waters, MA, United States) with an ACQUITY UPLC system coupled to the Waters Q-Tof Premier high resolution mass spectrometer. An ACQUITY UPLC BEH C18 column (2.1 mm × 50 mm) was used for chromatography analysis. Solution A was composed of 0.01% formic acid in water; solution B was composed of 0.01% formic acid in CH_3_OH. A linear gradient elution from 10 to 100% B over 10 min at 0.4 ml/min, 100% B over 3 min and re-equilibrated with 10% B for an additional 3 min. The injection volume was 1 μl. The entire column elute was introduced into the Q-Tof mass spectrometer. Ion detection was achieved in ESI mode using a source capillary voltage of 1.5 kV, source temperature of 120°C, desolvation temperature of 350°C, cone gas flow of 50 L/h (N_2_), and desolvation gas flow of 600 L/h (N_2_). Presence of the peak in the bacterial extract showing the accurate mass of ralsolamycin (*m*/*z* 1291.7142) was identified, and the corresponding peak areas from wild-type and mutant were compared and calculated.

## Results

### Expression of the *rmy* Genes and Production of Ralsolamycin Are Controlled by the PhcBSR QS System

**FIGURE 1:**
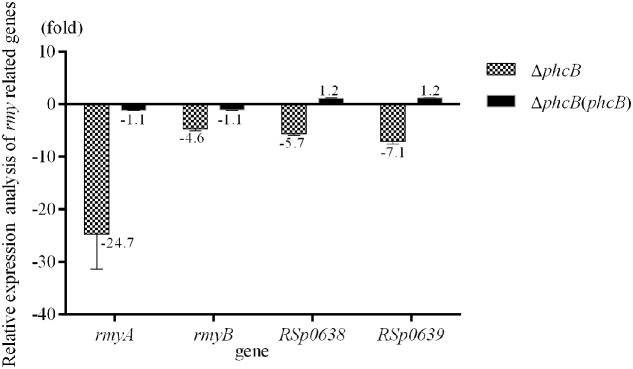
Quantitative Real-Time PCR (qRT-PCR) analysis of *rmy* clusters in *phcB* deletion mutant of strain GMI1000. The results were calculated as –ΔΔCt = –(ΔCt_mutant_ – ΔCt_wt_). Three biological repeats (independent cultures) and three technical repeats were done to calculate and compare the values, all statistics were presented as means ± SE.

**FIGURE 2:**
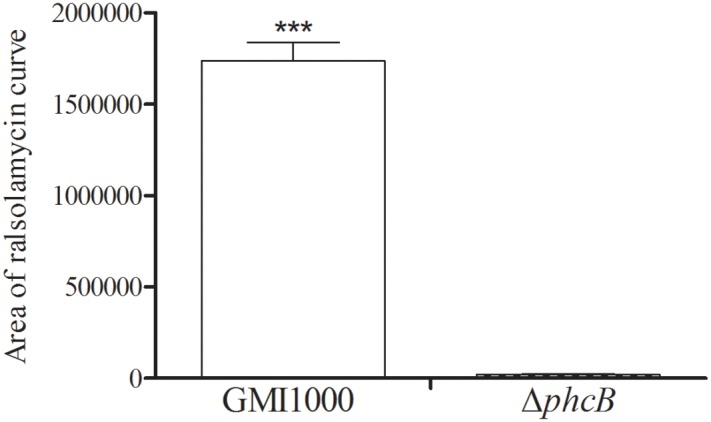
LC-MS analysis of ralsolamycin in the ethyl acetate extracts of the wide-type strain GMI1000 and *phcB* deletion mutant supernatant. The bacterial growth (under shaking) has been followed during 24 h by measurement of OD_600_ (1.5). The *p*-values from the Welch *t*-test comparing the production of wide type strain GMI1000 with the production of the Δ*phcB* strain (^∗∗∗^ *p*-value < 0.001).

To evaluate the role of the PhcBSR QS system in regulation of the *rmy* genes expression and ralsolamycin production, the QS signal synthase gene *phcB* deletion mutant was constructed in the genetic background of *R. solanacearum* strain GMI1000 by deletion of its coding sequence. The resulting strain Δ*phcB* was non-mucoid and nearly avirulent with weak cellulose activity, but was highly motile and with an increased polygalacturonase activity as previously reported (Flavier et al., 1997). The expression levels of *rmyA* and *rmyB* in Δ*phcB* were determined and compared to its parental strain GMI1000. In the Δ*phcB*, the *rmyA* transcription was drastically decreased by about 24-fold (Welch *t*-test, *p*-value < 3.64e-4; **Figure 1**), and the gene *rmyB* transcription was decreased by around 4.6-fold (Welch *t*-test, *p*-value < 3.02e-3; **Figure 1**). Meanwhile, deletion of *phcB* also resulted in decreasing the expression level of *RSp0638* and *RSp0639* by approximately 6- and 7-fold, respectively (Welch *t*-test, *p*-value < 2.32e-3 and 2.08e-3, respectively; **Figure 1**), which are the two neighbor genes of the *rmy* operon. *In trans* expression of a wild-type *phcB* gene in Δ*phcB* restored their expression to the wild-type levels. Consistent with the results of transcriptional assay, LC-MS analysis of the ethyl acetate extracts from wide-type strain GMI1000 and Δ*phcB* showed that ralsolamycin production in Δ*phcB* was decreased by about 81-fold compared with the wide-type strain (Welch *t*-test, *p*-value < 6.86e-5; **Figure 2**).

### Cell Density Affects the Expression Level of the *rmy* Genes

**FIGURE 3:**
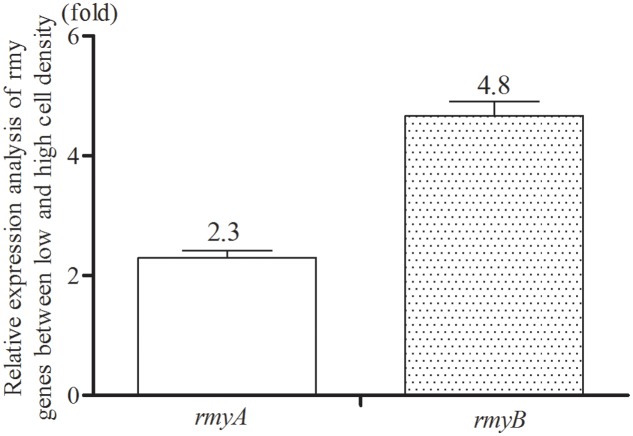
Quantitative Real-Time PCR analyses of *rmyA* and *rmyB* at high and low cell density. The results were calculated as –ΔΔCt = –(ΔCt_highcelldensity_ – ΔCt_lowcelldensity_). Three biological repeats (indedendent cultures) and three technical repeats were done to calculate and compare the values, all statistics were presented as means ± SE.

Given that bacterial QS systems modulate target gene expression in a population density dependent manner, the transcriptional levels of *rmyA* and *rmyB* in strain GMI1000 was then determined at different growth stages of bacterial cells. The results showed that the transcripts level of *rmyA* and *rmyB* in strain GMI1000 at a high cell density (∼10^8^ CFU/ml) was increased by about 2.3- and 4.8-fold, respectively (Welch *t*-test, *p*-value < 3.22e-3 and 2.18e-3, respectively; **Figure 3**), compared with those at a low cell density (∼10^6^ CFU/ml).

### Mutation of *phcB* Attenuates the Bacterial Induction Activity on FOC Chlamydospore Formation and Inhibitory Ability on *S. scitamineum* Growth

**FIGURE 4:**
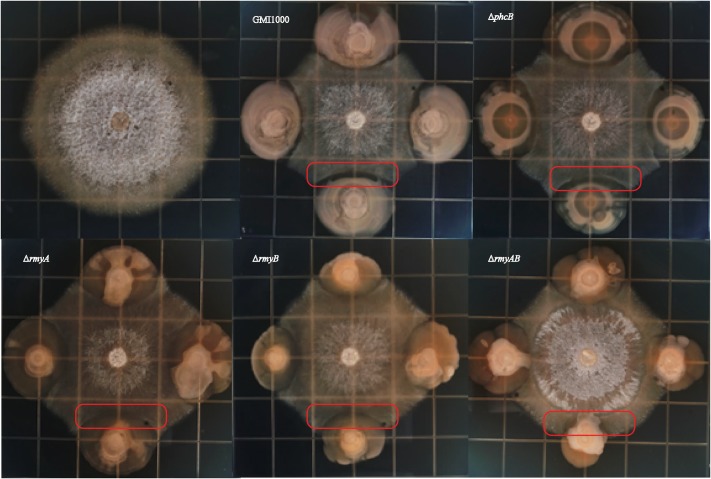
Bacteria-fungi interaction assay between *Ralstonia solanacearum* strain GMI1000 and XJZ2.

**FIGURE 5:**
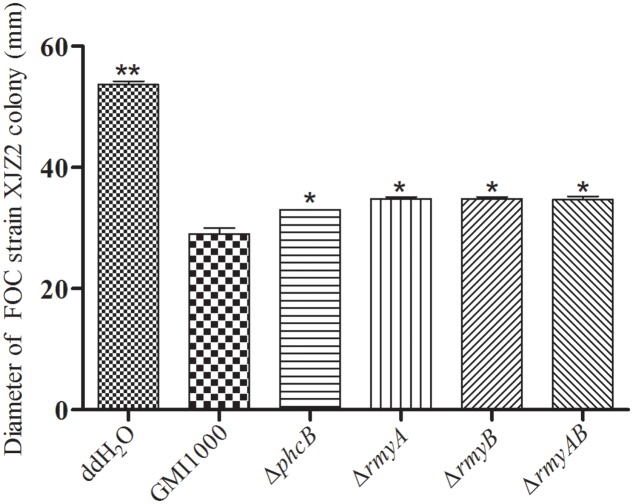
Diameter of XJZ2 colony co-culture with GMI1000 and its derivatives. Three biological repeats were conducted per strain. Bars indicate standard deviation (Welch *t*-test, comparison with the wide type strain GMI1000, ^∗^*p*-value < 0.05, ^∗∗^ *p*-value < 0.01).

Interaction of *R. solanacearum* strain GMI1000 and fungal pathogen FOC strain XJZ2 were examined by spotting them side by side in the same plate as shown in **Figure 4**. After 8 days of co-culture, distinct zones of fungal inhibition were formed in the area containing *R. solanacearum* wide-type strain GMI1000. We also detected the diameter of XJZ2 colony co-cultured with GMI1000 and its derivatives to quantify the inhibition effect, result demonstrated that the XJZ2 colony co-cultured with wide-type strain GMI1000 with the smallest diameter. In comparison to GMI1000, the reduction of *phcB* deletion mutant inhibition effect reached significant level (Welch *t*-test, *p*-value = 0.02; **Figure 5**). In addition, the chlamydospores of strain XJZ2 were routinely found in the interaction zones with wide type strain GMI1000 Supplementary Information File 2 (SI 2), whereas in the area containing *phcB* deletion mutant Δ*phcB*, chlamydospores were hardly found. Complementation of the mutant Δ*phcB* with a wild-type *phcB* gene restored the bacterial induction ability on chlamydospore formation Supplementary Information File 2 (SI 2). When the mutant Δ*rmyA*, or Δ*rmyB*, or the double deletion mutant Δ*rmyAB* was co-cultured with the fungal strain XJZ2, the chlamydospore was also hardly found within their interaction zones Supplementary Information File 2 (SI 2).

**FIGURE 6:**
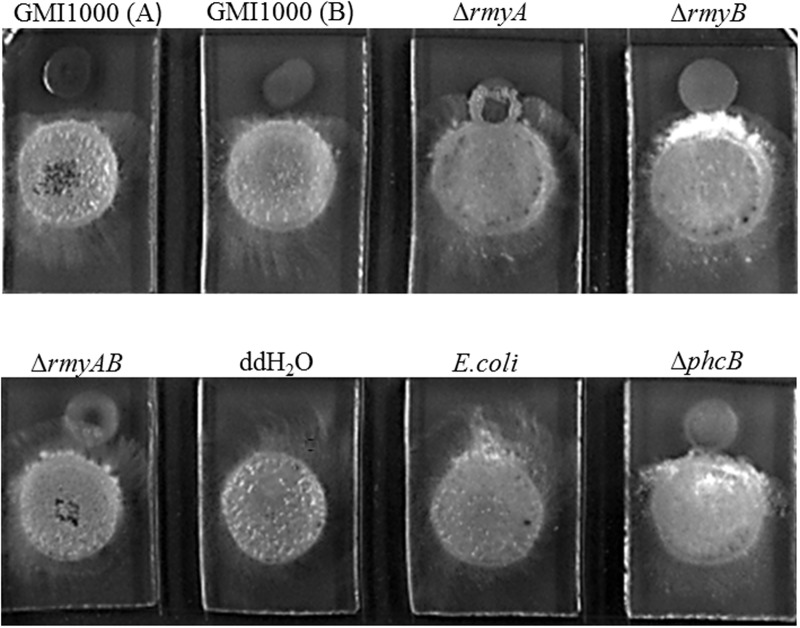
Co-culture experiments between *R. solanacearum* and *Sporisorium scitamineum*. GMI1000 (A) was cultured 24 h before adding the GMI1000 (B), other GMI1000 derivatives, and *MAT-1* and *MAT-2* mating mixture of *S. scitamineum*.

Distinct inhibition zones were also found between wide-type strain GMI1000 and the sugarcane fungal pathogen *S. scitamineum* (**Figure 6**). The colony of strain GMI1000 inoculated 1 day earlier generated a more obvious inhibition zones than the bacterial colony inoculated at the same time with the fungal strain, suggesting a clear dosage effect of the inhibitory compound(s) produced by strain GMI1000. The mutant Δ*phcB* could also inhibit the growth of *S. scitamineum*, but was weaker than the wide-type. In contrast, deletion of *rmyA*, *rmyB*, or *rmyAB* completely abolished the growth inhibition ability on *S. scitamineum*, as the hyphe of *S. scitamineum* could grow around or cover the colonies of these *rmy* gene deletion mutants, and the *E. coli* as a control showed no inhibition effect on the fungi.

## Discussion

Quorum sensing system is a widely conserved mechanism of bacterial cell-cell communications, which acts in a coordinated manner, and the individual bacteria can benefit from group behavior in competition for survive and persistence in nature (Fuqua et al., 1994; Von-Bodman et al., 2003). In *R. solanacearum*, the PhcBSR QS system serves as a master regulator to regulate most of the traits needed for infection and virulence (Denny, 1995, 2000; Schell, 2000). As an inter-species communication signal associated with competition for environmental niches, we are curious whether ralsolamycin biosynthesis is regulated by QS mechanisms. In this study, we provide sufficient genetic and chemical evidences that the two *rmy* genes associated with ralsolamycin biosynthesis and production in *R. solanacearum* are positively regulated by the PhcBSR QS system. During the course of bacteria–fungi interaction, we also showed that deletion of *phcB* attenuated the bacterial induction activity on chlamydospore development in fungal organisms, and reduced the inhibition activity to fungi.

It was predicted that up to five genes are involved in biosynthesis of ralsolamycin biosynthesis and transport, including *rmyA*, *rmyB*, *RSp0638*, *RSp0639*, and *RSp0640* (Spraker et al., 2016). Although the later three genes are not in the same operon as *rmyA* and *rmyB*, their protein characteristics, and the similar QS-dependent expression pattern as the *rmy* genes unveiled in this study seem to support that these genes are functionally related probably, for deleting *phcB* also resulted in significant reduction in the transcripts levels of the two genes (*RSp0638* and *RSp0639*) next to the *rmy* cluster.

Comparing with the NRPs that have been intensively investigated (Stachelhaus and Marahiel, 1995; Marahiel et al., 1997), the *rmy* genes in *R. solanacearum* GMI1000 are highly related to the syringomycin synthetase gene, which is required for the production of syringomycin in *P. syringae* (Bender et al., 1999). Another two NRPs product nunamycin and nunapeptin isolated from *P. fluorescens* In*5* are the key biocontrol components against *Rhizoctonia solani* (Michelsen et al., 2015). A BLAST search found that RmyA and RmyB are also similar to AmbB and AmbE associated with IQS signal biosynthesis in human pathogen *P. aeruginosa* (Lee et al., 2013), with over 74% coverage and 35% identity at amino acids level. For the phosphate depletion is a host stress signal commonly encountered by invading pathogen *P. aeruginosa* (Lee et al., 2013), which actives the IQS system. Moreover, evidence reveals that EfpR is a novel key component of the complex regulatory network of the *R. solanacearum* cell, tightly linking the bacterial metabolism to virulence in response to multiple environmental signals (Perrier et al., 2016). Accordingly, whether there is any stress or environmental signal will involve in modulating the ralsolamycin production is still worth for further research.

*Ralstonia solanacearum* is known to wide distributed and persisted in soils for remarkably long periods (Alvarez et al., 2010), and the soil is a heterogeneous and complex microcosm replete with inter-organismal interactions, thus, the *R. solanacearum* will encounter a diversity of other soil microbes. The FOC and *S. scitamineum* are two spread worldwide and causes considerable yield losses and reduction in their host plants (Singh et al., 2004; O’Donnell et al., 2009). Based on our results, the ralsolamycin functioned in the crosstalk between *R. solanacearum* and the two tested fungi. On one side, the *R. solanacearum* can use the ralsolamycin to antagonize the fungi; on the other hand, which can also induce the fungi around to formate chlamydospores significantly, together with the fact that *R. solanacearum* can enter the endofungal lifestyle with chlamydospores (Spraker et al., 2016), which is a novel persistence mechanism for bacterial survival in the harsh environments. Moreover, we also want to determine whether the ralsolamycin can affect the sexual matting of *S. scitamineum*, result showed that except for the antagonization on *S. scitamineum*, the ralsolamycin does not inhibit the sexual matting. It was noteworthy that there was still weak inhibition effects on the fungi after deleting the *rmy* genes, thus, maybe some other metabolisms are also with the antagonization effect on the fungi. Obviously, the ralsolamycin plays a major role. It has been demonstrated that the “trade-off” existed between virulence factor production and bacterial proliferation is controlled by PhcBSR system dependent regulatory protein PhcA, a *phcA* mutant has an expanded metabolic versatility to metabolize up to 17 substrates and proliferation ability more than the wild-type (Peyraud et al., 2016). Rather paradoxically, our results indicate that the production of ralsolamycin is decreased dramatically when *phcB* is inactive. Hence, we can hypothese that the *R. solanacearum* can compete for a regnant niche and enhance survival ability for the partnering fungus under the modulation of PhcBSR QS system, and it shall be one kind of active regulatory mechanism to adapt to the harsh environment when the *phcB* is active, which may also contribute to understand why the *R. solanacearum* species complex could share such a broad ecological range with various host plants and soil cohabitants.

## Conclusion

We have demonstrated that the biosynthesis of inter-kingdom communication signal ralsolamycin is regulated by the *phcB* dependent QS system. Significantly, the findings from this study unveiled a link between QS and inter-kingdom communication. Further studies are required to understand the molecular mechanisms and signaling pathways that govern the QS-dependent expression of the *rmy* genes and ralsolamycin.

## Author Contributions

PL, YD, and L-HZ designed the experiments and wrote the paper; WY, JY, YC, JZ, and ML helped to obtain the mutants; SF and SS helped to perform the LC-MS analysis; YD and L-HZ revised the manuscript.

## Conflict of Interest Statement

The authors declare that the research was conducted in the absence of any commercial or financial relationships that could be construed as a potential conflict of interest.
