# Supplementary material for: Modulation of Inter-kingdom Communication by PhcBSR Quorum Sensing System in Ralstonia solanacearum Phylotype I Strain GMI1000
Source: Front Microbiol. 2017 Jun 23;8:1172. doi: 10.3389/fmicb.2017.01172 (PMC5481312; doi:10.3389/fmicb.2017.01172)

SI 1 Gel electrophoresis results of *phcB* and *rmy* deletion mutants. The bands from left to right were 1, Δ*phcB* (EP1); 2, Δ*phcB* (GMI1000);3, Marker 5k, (bands from top to bottom were 5k, 3k, 2k, 1.5k, 1k, 750bp, 500bp, 250bp, 100bp); 4,wide type stain (WT, GMI1000); 5 WT (EP1); 1, 2, 4, 5 were amplified by the primer pair *phcB*-L1/R2; 6, complementary strain of Δ*phcB* (GMI1000); 7, complementary strain of Δ*phcB* (EP1); 6, 7 were amplified by the primer pair *phcB*-CF/CR; 8, Δ*rmyA* (GMI1000) amplified by the primer pair *rmyA*-L1/R2; 9, Δ*rmyB* (GMI1000) amplified by the primer pair *rmyB*-L1/R2; 10, WT (GMI1000) amplified by the primer pair *rmyA*-L1/R2; 11, WT (GMI1000) amplified by the primer pair *rmyB*-L1/R2.

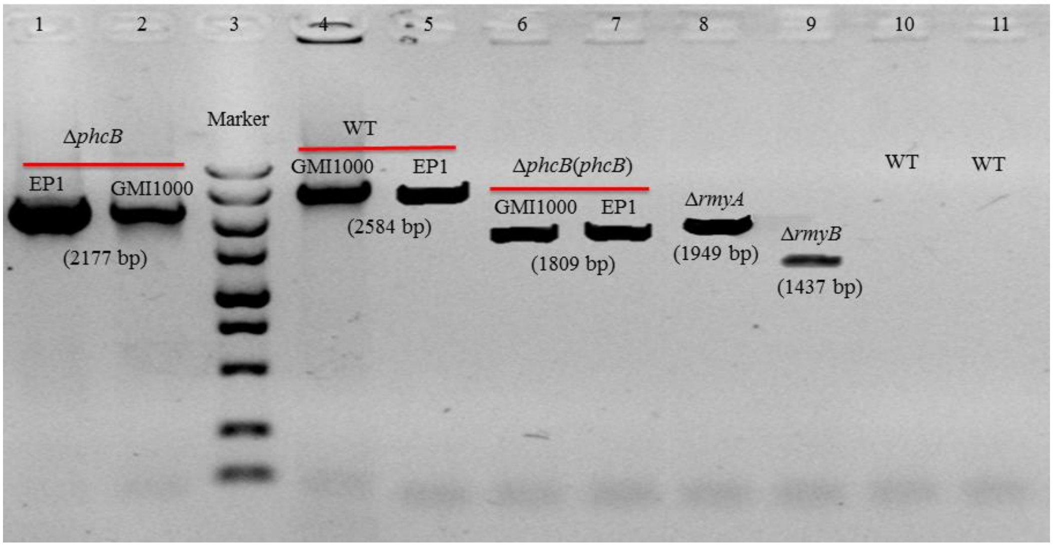

Supplement: Supplementary file 1 [file Image_1.PDF]
