# Supplementary material for: Modulation of Inter-kingdom Communication by PhcBSR Quorum Sensing System in Ralstonia solanacearum Phylotype I Strain GMI1000
Source: Front Microbiol. 2017 Jun 23;8:1172. doi: 10.3389/fmicb.2017.01172 (PMC5481312; doi:10.3389/fmicb.2017.01172)
Supplement: Supplementary file 2 [file Image_2.PDF]

SI 2 Detection of XJZ2 chlamydospores induced by wide type GMI1000 and derivatives. (A) to (G) represent sterile water, wide type GMI1000, Δ*phcB*, Δ*rmyA*, Δ*rmyB*, Δ*rmyA*/*B*, and Δ*phcB* (*phcB*) respectively.

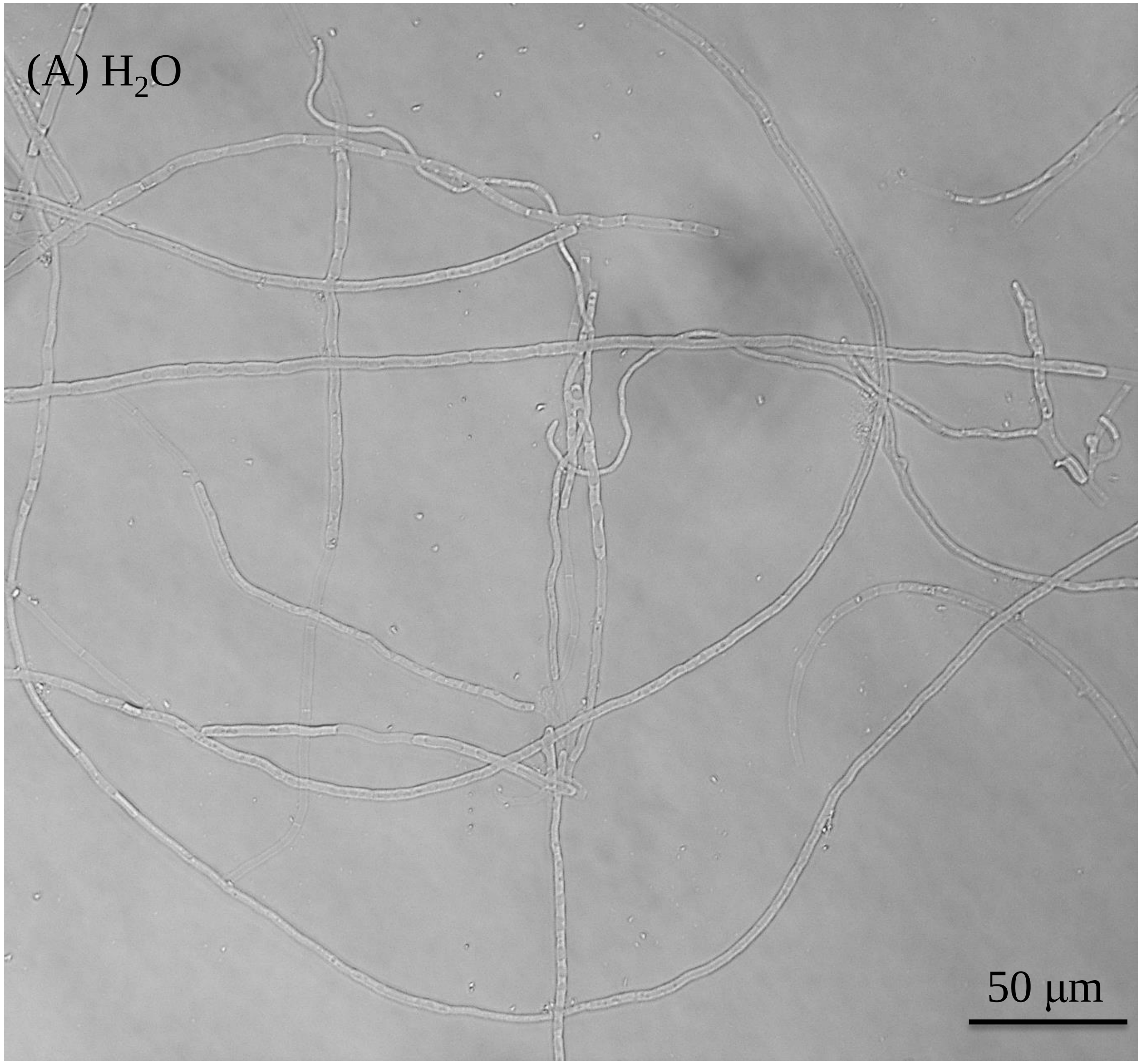

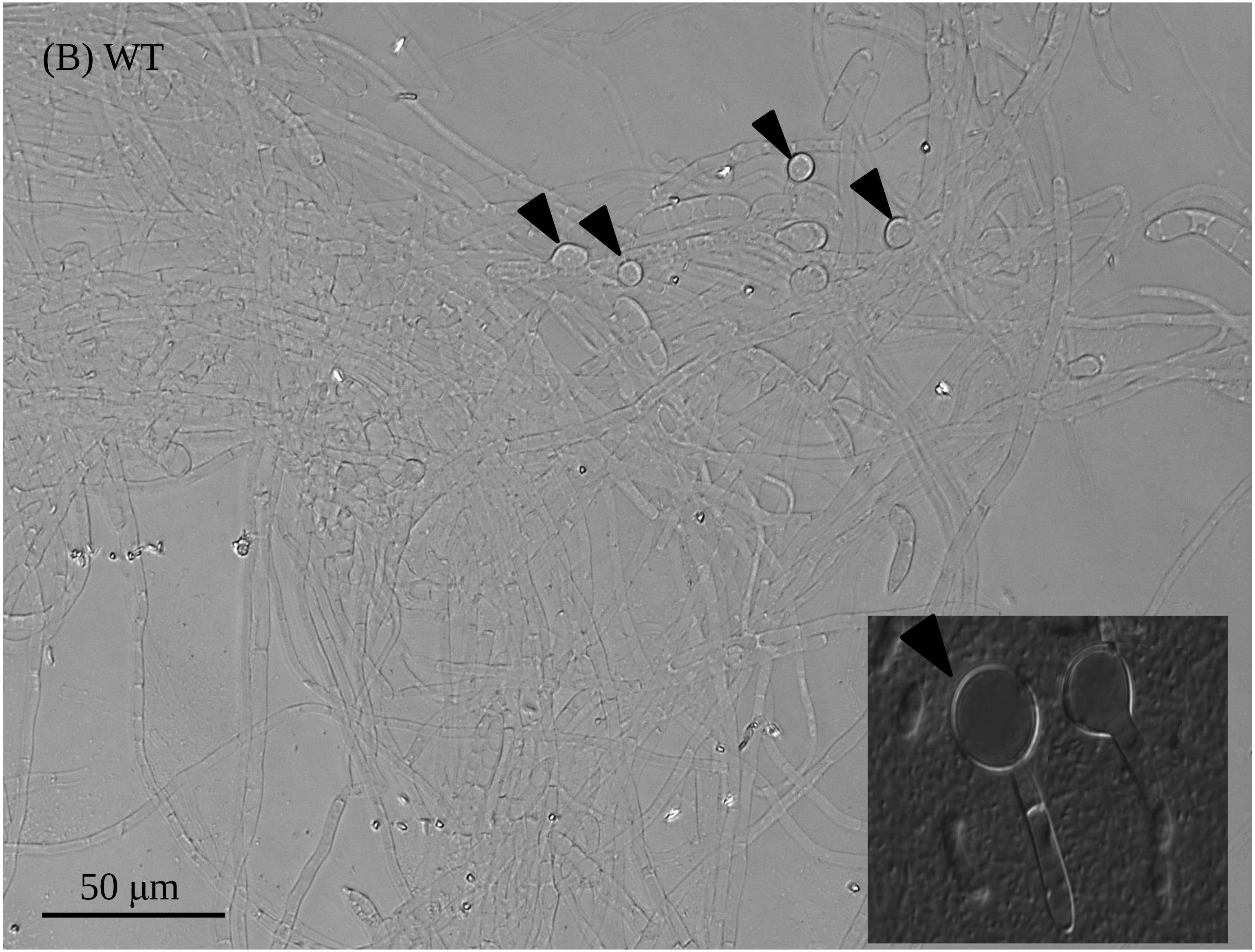
(B) WT
50 µm

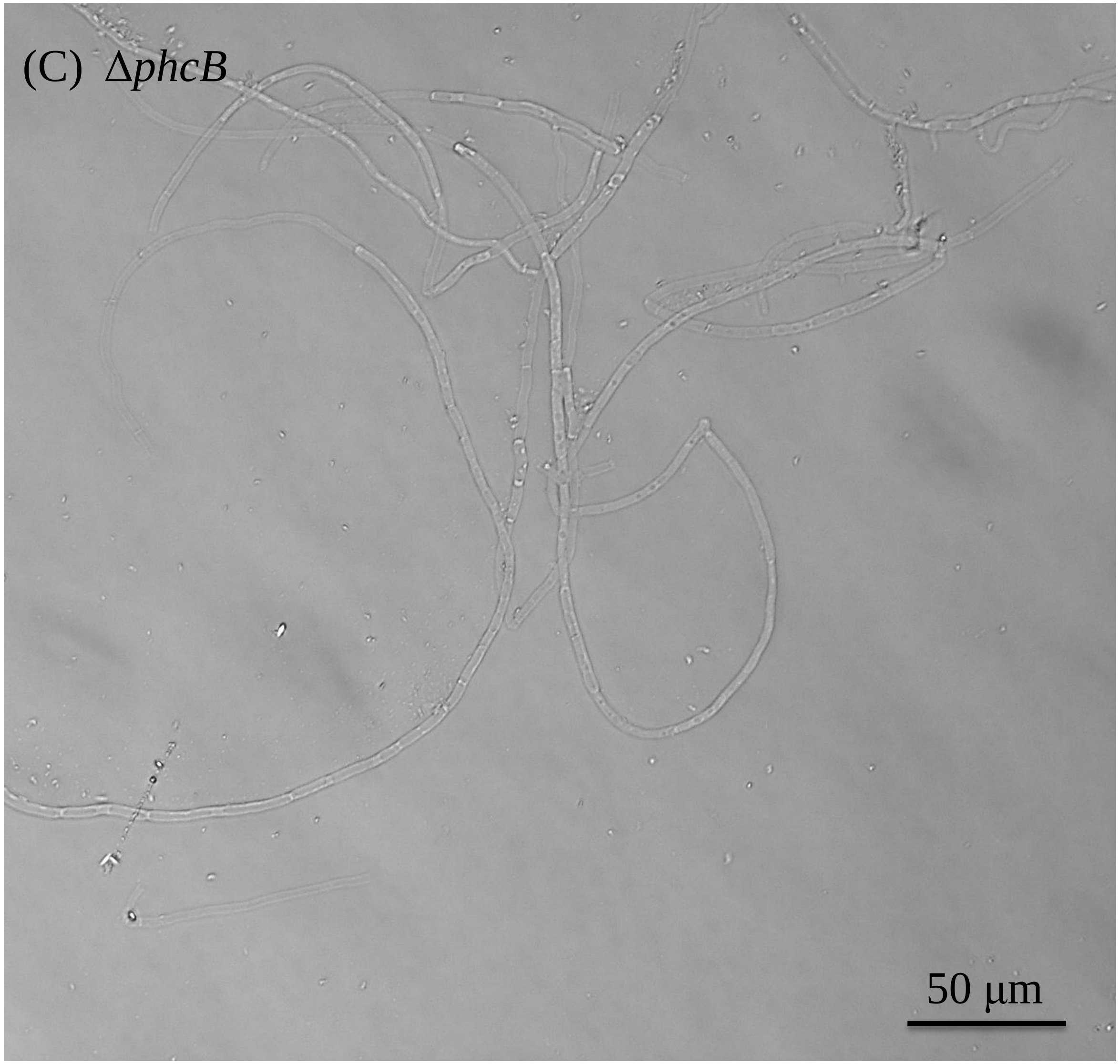
(C) ΔphcB
50 µm

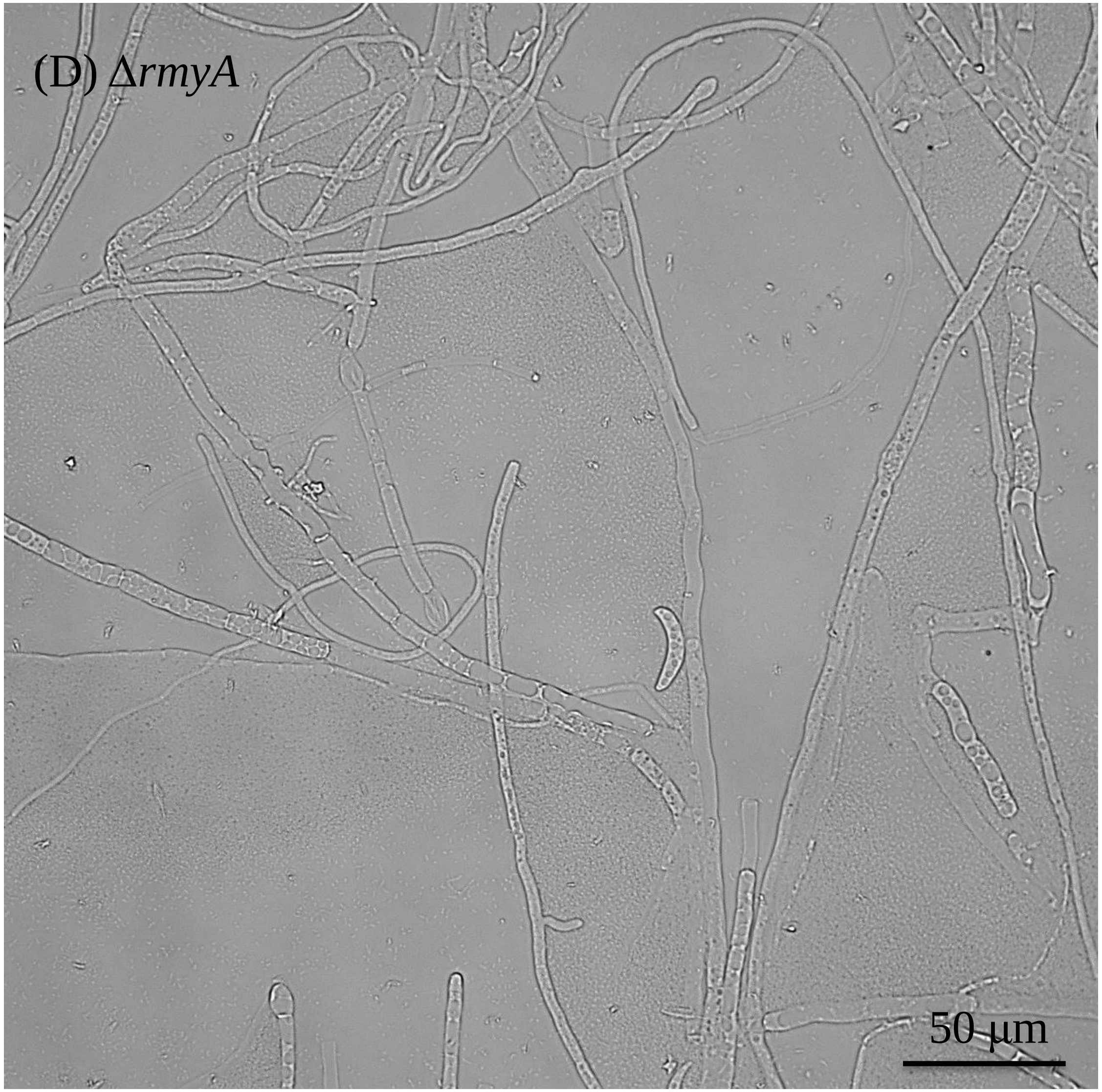
(D) Δ*rmyA*
50 μm

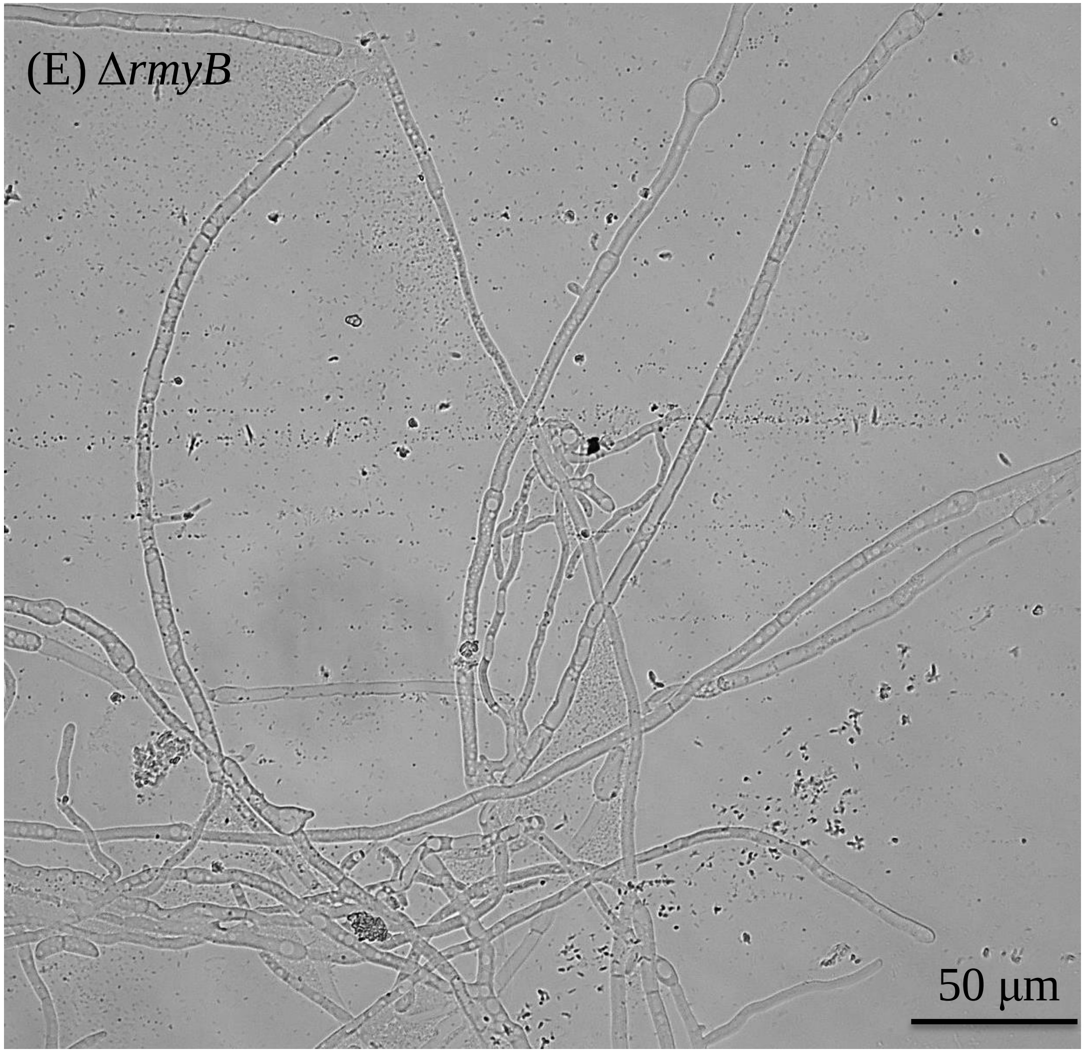
(E) Δ*rmyB*
50 μm

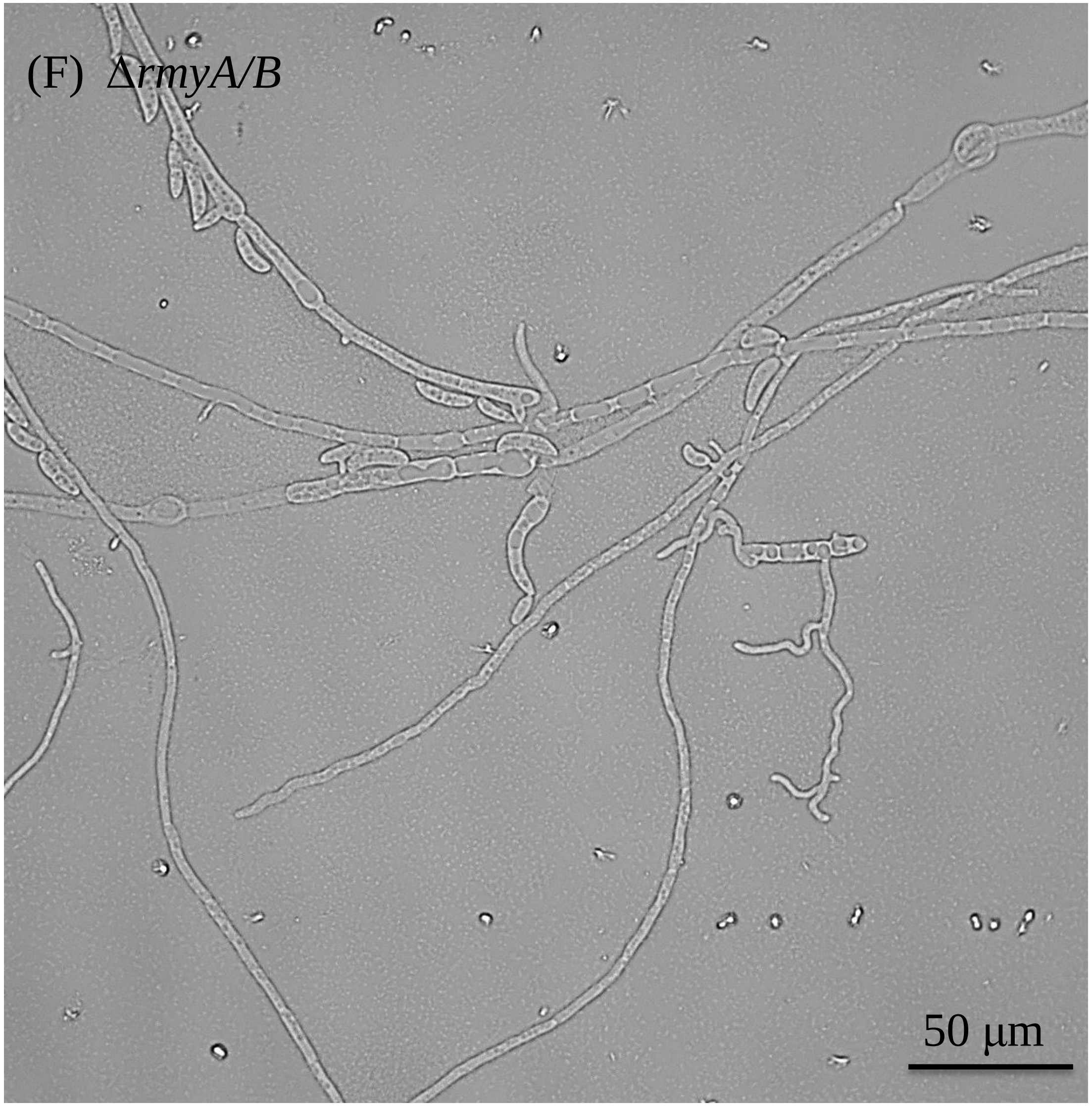
(F) Δ*rmyA/B*
50 μm

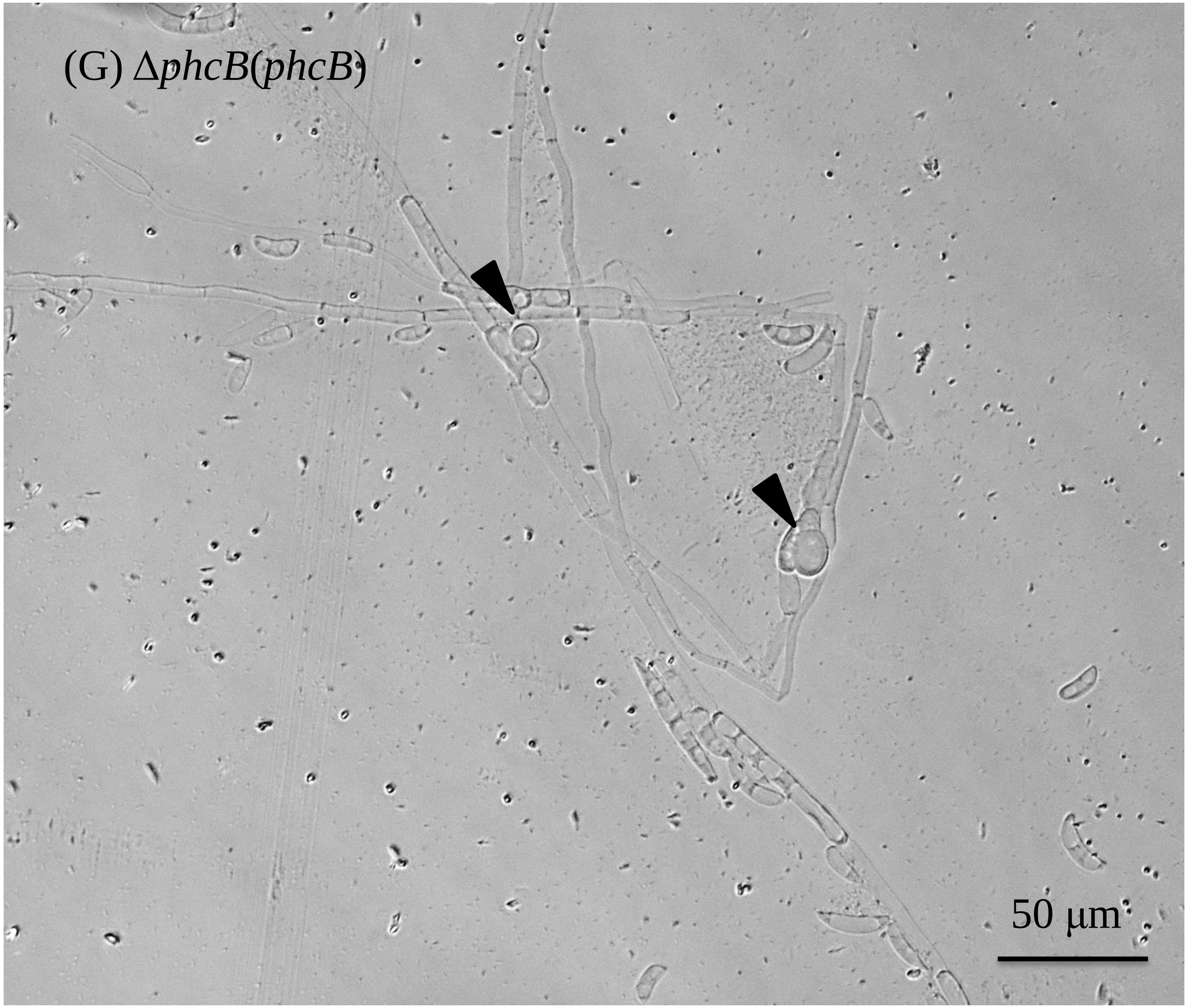
(G) Δ*phcB*(*phcB*)
50 µm
